# A qualitative investigation into key cultural factors that support abstinence or responsible drinking amongst some Pacific youth living in New Zealand

**DOI:** 10.1186/1477-7517-9-36

**Published:** 2012-08-16

**Authors:** Tamasailau Suaalii-Sauni, Kathleen Seataoai Samu, Lucy Dunbar, Justin Pulford, Amanda Wheeler

**Affiliations:** 1 Clinical Research and Resource Centre, Waitemata District Health Board, Level 3, Snelgar Building, Private Bag 93115, Henderson, Waitakere, 0650, New Zealand; 2 Vaaomanu Pasifika Unit, Victoria University of Wellington, Wellington, New Zealand

**Keywords:** Pacific peoples, Alcohol, Youth, Risk, Drinking

## Abstract

**Background:**

Abstinence and responsible drinking are not typically associated with youth drinking culture. Amongst Pacific youth in New Zealand there are high numbers, compared to the general New Zealand population, who choose not to consume alcohol. The Pacific youth population is made up of several ethnic groups; their ethno-cultural values are largely Polynesian and heavily influenced by the socio-economic realities of living in New Zealand. This paper explores factors that support abstinence or responsible drinking amongst Pacific youth living in Auckland.

**Methods:**

A qualitative study comprised of a series of ethnically-, age-, and gender-matched semi-structured focus group discussions with 69 Pacific youth, aged 15-25 years from a university and selected high-schools. Participants were purposively sampled.

**Results:**

Key cultural factors that contributed to whether Pacific youth participants were abstinent or responsible drinkers were: significant experiences within Pacific family environments (e.g. young person directly links their decision about alcohol consumption to a positive or negative role model); awareness of the belief that their actions as children of Pacific parents affects the reputation and standing of their Pacific family and community (e.g. church); awareness of traditional Pacific values of respect, reciprocity and cultural taboos (e.g. male–female socialising); commitment to no-alcohol teachings of church or religious faith; having peer support and experiences that force them to consider negative effects of excessive alcohol consumption; and personal awareness that being part of an (excessive) drinking culture may seriously affect health or impede career aspirations.

**Conclusions:**

The narratives offered by Pacific young people highlighted three key communities of influence: family (immediate and extended, but especially siblings), peers and church. Young people negotiated through these communities of influence their decisions whether to drink alcohol, drink excessively or not at all. For each young person the way in which those three communities came together to support their decisions depended on the specificities of their lived contexts. Pacific young people live lives that share some things in common with other New Zealand youth and others which are more specific to a Pacific ethnic group, especially in relation to the traditional beliefs of their Pacific parents and community. In the development of alcohol harm reduction strategies seeking active Pacific young person and family compliance, it is these “other ethnic things” that requires careful and more qualitative consideration.

## Introduction

Young people are as diverse as their societies. Various influences both positively and negatively impact on their behaviours and sense of self. These influences are both internal and external and influence young people’s choices when faced with whether to engage in risky health behaviours (such as alcohol misuse) or not, or whether to make positive health choices (such as abstinence or responsible alcohol drinking) or not.

In New Zealand, the youth alcohol drinking culture is of concern. In 2010 there has been an increase in media concern about youth mortality associated with alcohol use [1,2]. Costs associated with risky youth alcohol use was estimated to be $6.5 billion in social costs (in 2005/06) [3]. In a recent New Zealand health study, 16–24 year olds overall experienced higher levels of harmful effects from their alcohol use than people in older age groups [4]. This age group can be further analysed by ethnicity.

Ethnicity is used by Statistics New Zealand to categorise and name those people who self-affiliate with others who share cultural characteristics such as “a common proper name; one or more elements of common culture…which may include religion, customs or language; unique community of interests, feelings and actions; a shared sense of common origins or ancestry; and a common geographic origin” [5]. The ‘Pacific’ ethnic label has been more or less used to describe peoples who self-affiliate with one or more of the island nations in the Pacific and their cultural origins. Although other Pacific island nations are implicitly included within the label, the main island ethnic cultures that come together under this label in New Zealand are those who share a predominantly Polynesian origin, i.e. people from Samoa, Tonga, Fiji, Cook Islands, Niue, Tokelau, Tuvalu and Tahiti. More recently peoples and cultures from Melanesia (such as from Papua New Guinea, Solomon Islands and Vanuatu) and Micronesia (such as Guam) are being publicly recognised under the label, although their population numbers are comparatively very small. Politically in Aotearoa New Zealand, Māori as the indigenous people or *tangata whenua*, are excluded from the Pacific label even though technically, in terms of ethno-cultural values, they are part of the Polynesian family.

New Zealand is today home to approximately 266,000 people who self-affiliate as being of Pacific ethnicity [6]. In 2006 they made up 6.9 % of the total New Zealand population and are predicted to make up 10 % by 2026 [7]. The New Zealand Statistics ethnicity classification system counts Pacific peoples who record up to six different ethnic affiliations [5]. The New Zealand youth population is more ethnically diverse than the total population. In 2006 Pacific young people made up 9.3 % of the total New Zealand youth population [8]. Most of the Pacific population, young and old, live in urban centres, mostly in the greater Auckland area.

Like other young people, Pacific young people develop a sense of self and self responsibility through gaining a sense of identity and belonging to the different groups who share their social, economic and political milieu, especially their families, peers and churches [9-11]. These groups share a common set of beliefs and values which are often evident in what their members say and do. This shared belief and value system may also be described as a cultural value system, the ethnic aspects of which stem from the peculiarities of a unique language, socio-political structure, traditions, shared ancestry and ancestral connections to land. While these ethnic values or aspects are most obviously captured by ethnic specific language terms, the ways in which ethnic communities behave can also capture and reflect ethnic nuances. In ethnic diasporic communities these characteristics may be subtly expressed or expressed in hybrid form and, as some have argued [12,13], this hybrid form reflects a sub-culture that retains key elements of the dominant ethnic culture, rather than creating a new and different ethnic culture. In other words, it is possible to trace the continuing influence – or lack thereof – of key ethnic or cultural values in the way in which young Pacific people in New Zealand behave and talk about what matters or not to them, even if this is expressed using a foreign tongue [10,13,14]. Given that according to the 2006 Census New Zealand-based Pacific young people speak English more than their respective Pacific mother tongue, the use of a hybrid English-Pacific language to express their identities is more than likely to be the case for many Pacific young people growing up in New Zealand [9,15-18].

A large percentage of Pacific young people in New Zealand also come from families who affiliate with at least one religious group. Census 2006 statistics indicate that 83 % of Pacific peoples recorded having at least one religious affiliation; and almost all (97 %) of these identifying with at least one Christian religion [19]. This is not surprising given that their Pacific homelands are highly Christian communities also. Church life for many of the New Zealand Pacific diaspora involves the whole family – parents and children. In the four largest Pacific Christian denominations in New Zealand (Catholic, Methodist, Congregational and Presbyterian churches) alcohol drinking is permitted and monitoring of consumption levels is varied.

The drinking cultures of Pacific peoples’ in New Zealand, including its youth, are characterised by drinking high levels of consumption by fewer occasions per annum, i.e. binge drinking, compared to the general New Zealand population [4,20,21]. This level of alcohol consumption places young Pacific drinkers at increased risk of experiencing alcohol related harm. There is, however, a large proportion of young people generally who choose not to consume alcohol, either at all or excessively [4,22]. The choice to abstain or drink responsibly is influenced by a range of factors or influences, including ethno-cultural values [17,18]. Understanding how these ethno-cultural values might be present in the talk of Pacific young people who choose to abstain or drink responsibly in New Zealand is a key step in determining how they process their decision/s to do so. This is a necessary background step to determining what ought to be prioritised in the development and operationalisation of harm reduction strategies for young Pacific people against risky alcohol related behaviours.

It is useful to note Oates et al’s [23] point that protective factors are not necessarily the converse of risk factors; that resilience factors are also sometimes used in exchange for protective factors; and that, as Rutter argues, these positive experiences while they in themselves may not “exert much of a protective effect, they can be helpful if they serve to neutralize some risk factors” (p.119) [24]. Each of these points need to be considered during the exercise of developing harm reduction strategies based on either the identification of risk or protective factors.

## Methods

Data were collected via a series of semi-structured focus groups during 2008. The focus group method enabled participants the opportunity to share top of mind views and experiences with others in the group, utilising the interactive dynamic of the group to simultaneously draw out peer and individual understandings of the topic [38]. At a practical level it also allowed the study to capture a larger number of participants within restricted time and resource constraints.

### Sample size and distribution

Participants for focus groups were purposively selected [38]. Focus groups were ethnically-, age- and gender-matched to minimise cultural barriers to participant interaction: of the 21 focus groups conducted 10 were male only and 11 were female only; 7 involved senior high school students aged 15-17 years and 14 were university students aged 18–25 years: 6 focus groups involved Samoan students; 6 were of Cook Islands students; 5 were of Tongan students; 3 were of Niuean students; and 1 was of a mixed Pacific and mixed Pacific and non-Pacific ethnicities group. For ease of access the age definition of Pacific youth was limited to the age group 17–25 years and recruited from either a high school or university. Ethnic specific focus groups were conducted for Samoan, Tongan, Cook Islands and Niuean participants, on the basis that they represented the most numerous Pacific populations in New Zealand [22]. In recognition of the number of Pacific peoples in New Zealand who self-affiliate as being of mixed ethnicities, a mixed ethnicity focus group was included. Participants of mixed Pacific ethnicities or of mixed Pacific and non-Pacific ethnicities were eligible to participate in the ‘mixed ethnicity’ focus group or with the single Pacific ethnicity group they identified with. This classification was implemented to capture those Pacific young people whose parents were of different ethnic ancestries and they self-identified with more than one of those Pacific ethnic groups. New Zealand Statistics allows New Zealand citizens to record an ethnicity combination output of up to three pan-ethnic groupings, such as Maori/Pacific peoples, Pacific peoples/Asian, Pacific peoples/European/Other Ethnicity, and so on [5].

The discussion groups were gender differentiated on the understanding that this would allow participants to be more open to sharing gender-sensitive information. Participants self-identified as abstainers (those who had not had an alcoholic drink in the last 12 months), or responsible drinkers (a guide to responsible drinking from the Alcohol Advisory Council of New Zealand for recommended maximums on a single, daily and weekly basis for both men and women was provided [25]).

### Participant recruitment and process

Participants in the 15–17 year age range were sought from selected high schools across the Auckland region. The research team approached a key person (including teachers, youth liaison and social workers) at each of the targeted schools to identify appropriate participants for the study. Participants in the 18–25 year age range were sought from the University of Auckland’s Centre for Pacific Studies (CPS). A key person at CPS assisted in the recruitment of appropriate (i.e. those who fit the selection criteria) university participants. Arrangements for suitable times and locations for focus group discussions with both age groups were made through these key people.

Potential participants were given an information sheet detailing the study’s aims, expectations and benefits/risks of participation and guidance about responsible drinking. Parents of the high school study population were informed about the research project and because the age of participation was 17 years of age (16 years is the legal age of consent in New Zealand) passive consent was obtained (i.e. opportunity was given to parents via the parent information sheet given to them to have their child withdraw themselves from the study if the parent wished). Those students who agreed to participate were invited to attend an appropriate focus group session. All focus groups were conducted in English and facilitated by a trained research assistant and where possible, focus group and facilitators were gender-matched.

Two sets of topic guides were used to elicit discussion: one for the abstinence group and another for the responsible drinking group. The fields of enquiry were: perceptions on alcohol; thoughts/views on the culture of drinking; the effects of alcohol on family; thoughts/views on the legal age of drinking; social and moral consequences of drinking; and key factors that support abstinence or responsible drinking. Emerging discussion areas included drinking cultures in Pacific homelands.

Prior to focus group discussions a brief verbal explanation of the study was given. Participants completed a demographic information sheet for recording purposes and gave written consent. Focus groups were audio recorded and then transcribed verbatim by Pacific research assistants. The study received ethical approval from the Health and Disability Northern Y Regional Ethics Committee (Ref: NTY/08/04/027).

The study adopted the Pacific research principles of respect (or *faaaloalo* – Samoan language term) and reciprocity (or *na veidinadinati ni veisolisoli* – Fijian language concept) in the practice of presenting a *meaalofa* (gift – Samoan language term) to participants in appreciation of their time and spirit of sharing, as advocated by the Pacific Health Research Guidelines [26].

### Analysis

Interview transcripts were read and re-read by the research team to identify key issues associated with responsible drinking behaviour or abstinence among Pacific youth. A general inductive approach [27] was used to identify common themes across the various group data. Themes were identified using key words that arose from the transcripts, words considered by the research team as reflective of ideas or thoughts raised by participants that when read in the context of the participant’s or group’s wider comments could be considered a key topic theme. These key words or themes were then used to code the qualitative narrative. This was done by two members of the research team. Some comparisons across age, gender and ethnic lines were made.

## Results

### The sample

Twenty-one focus groups were successfully completed with 69 participants (31 males and 38 females). The average age of participants was 18.5 years (SD 2.78; range 15–25). The majority of participants were born in New Zealand (71 %). For the 29 % of Pacific Island-born participants, the average length of time reported living in New Zealand was 9.9 years (SD 7.44; range 7 months to 22 years). The largest proportion of participants self-identified as mixed-Pacific ethnicity (31.9 %), followed by Samoan (29.0 %), Tongan (21.7 %), Cook Islands (11.6 %), and Niuean (2.8 %). Overall, there were relatively even numbers of self-identified abstainers (49 %) and responsible drinkers (51 %) represented in the study.

For anonymity, all participants are cited using the generic male identifier.

### Key themes arising

The key themes arising from the narratives of participants can be grouped into four main areas: 1. family environment; 2. ethno-cultural pride and values; 3. church influences; and 4. peer relationships and personal aspirations. These were the main themes raised throughout participant focus group narratives and are listed in no order of priority. Within these broad themes participants spoke about having significant experiences of a family environment where events affecting them, their parents, siblings and wider family members lend themselves to making the commitment they currently held to either not drink alcohol at all or not drink excessively. The idea of shame (on them, their families, churches and ethnic peoples) also permeated some of the narratives around why some of these Pacific young people chose to abstain or not drink irresponsibly. Church influence was often raised as a key institution for learning about the negative risks of alcohol consumption and as a site that young people still affiliated with, even if merely to transport family members to and from. The peer influence, both negative and positive, was also noted but interestingly the influence of schools were mentioned mainly in relation to being the site of peer pressure to drink rather than as a site for proactive learning about the risks of excessive alcohol consumption. Lastly, taking personal responsibility for the choices they make was a sub-theme that was emphasised by the young people of this study.

### Theme 1: Family environment

Participants talked broadly about four influential factors in relation to their family environment that contributed to their choices to abstain or drink responsibly: 1. parents and family role models who drank responsibly or who abstained and were happy; 2. parents and family role models who drank irresponsibly and created negative feelings; and 3. own fear of being reprimanded by parents.

#### Family environment: “Happy drinkers” versus “Heavy drinkers”

From our focus group data the idea of “happy drinkers” and “heavy drinkers” seem to capture well what in these Pacific young people’s words is a “cool” or “alright” drinking habit versus what is not a good drinking habit where a young person decides that he does not want to “follow in [their] footsteps”. In the two excerpts below from Focus Groups 8 and 9, the first offers the idea that alcohol drinking could be associated with “cool” family events where it helped to create “a happy buzz” in the household and where the cares of financial constraints seem forgotten, at least for the moment (Focus Group 8: Quote 1). In this situation the young person suggests that alcohol drinking can be something positive; that it can have positive consequences. He suggests that there can be and are in his family “happy drinkers”. He says:

“In my family like drinking…they have a couple of drinks. It’s a socialising thing and also creates a happy buzz. Like I said before that when my parents drink they’re like not stressed; like throughout the week and they get lots of money and they’re like “yaaayyy”. Like we have happy drinkers and they’re all in that buzz and then just like wanna sing songs bring out the uke bring out the spoons and then they sing all these songs and it’s like cool” (Focus group 8: Quote 1).

The next excerpt (Focus Group 9: Quote 2) talks about “heavy drinkers” in the context of “other family” members who drink heavily, so much so that the wives and children of these Uncles (who are the “heavy drinkers”) seem, at least to the young person sharing his views, to be a burden on them. This young person draws comparisons between his non-alcohol drinking parents and those of his cousins and decides that he is “lucky [his] dad’s not like that”.

“My parents don’t drink and stuff so because of their morals they’ve taught me and stuff it’s kinda (sic) put me in the right path. I have other family that drink as well and I could also see the differences coz …I could see like with my aunty and my uncles and that, like my uncles would be the heavy drinkers and my aunties would be there looking after the kids and stuff. And I don’t tend to see them like helping out around the house stuff and I was just thinking you know lucky my dad’s not like that and my mum…yeah. Well my parents have a big part in it…” (Focus group 9: Quote 2).

These Pacific young people suggest that in processing whether or not they will drink, drink too much or not drink alcohol at all, the drinking habits and practices of the adults in their households or wider family circles do impact on their decision-making processes. The impact of parents on young people’s choices is raised explicitly throughout the different focus groups. In the two excerpts from two different focus groups cited immediately below (Quotes 3 and 4), the point is raised emphatically.

*“Well my biological father, he’s an alcoholic…he currently has liver failure. He could consume a whole bottle of vodka without any mix, he’ll just drink it straight and he could at least drink a bottle a day. My mum said it’s not really a good thing. That’s why she’s always cautious of me of drinking as well…she’s always scared that* [I might] *go down that route but nah, I don’t drink like that… I guess that* [my father being an alcoholic] *definitely would have an impact…like I certainly did not wanna go on that road and follow in his foot steps…” (Focus group 16: Quote 3).*

*“*[My father’s drinking affected] *the whole family. Coz he used to go after anyone and everyone. And he like jumped out of the car in the middle of the motorway while my mum was driving and ran across like yelling at people and stuff and I was like* [afraid]*…he used to scare like the crap out of me coz… one time I saw him and like he was like getting held down by 10 police officers and yeah like my dad’s a Palagi [European] so like you don’t expect that from Palagis but they’re just as bad as Islanders and Maoris” (Focus Group 6: Quote 4).*

Indeed the young person from Focus group 12 whose voice is quoted in the next excerpt (Quote 5) suggests that it is the primary responsibility of parents to teach their children “in the beginning” how to drink responsibly.

*“If you’re taught…the good and bad stuff about everything then… you can like weigh up your* [options]*…everyone has their own limits; like some people can drink responsibly and they know when to stop, but others don’t know when to stop… so if you’re taught in the beginning…you can decide for yourself, like when to drink and how much to drink and that’s cool” (Focus group 12: Quote 5).*

Another Pacific young person from focus group 13 offers us insight into his process for assessing – which is quite careful and sensitive – the pros and cons of different situations which Pacific young people find themselves with regards to the influence of parents who are “happy drinkers” versus “heavy drinkers” on whether or not they should drink. He suggests at Quote 6 below that the fact that his father didn’t drink and seemed “alright” influenced him “to not want to drink”. The implication is that where there are positive consequences to decisions not to drink for those adults of significance to young people and this is witnessed by them, any pressure to drink and especially to drink irresponsibly and excessively can be lessened by this experience.

“I think my dad not drinking, influences me to not want to drink. Coz I don’t know, maybe people with parents who do drink or do have an alcohol addiction like maybe they feel like they need that as well. But um, yeah, my dad doesn’t drink and he’s alright. So, I feel that I’ll be alright if I don’t drink as well” (Focus group 13 – Quote 6).

The young people of our study also acknowledged that “heavy drinkers” may also drink heavily because they are depressed and wish to forget a traumatic event and “their sorrows”. The two excerpts below (Quotes 7 and 8) highlight two such situations.

“I think people drink because they drink their sorrows away. Um you know they drink because they’re depressed and if something’s gone wrong in their life, it’s, you know, they turn to drinking. It’s yeah um what do you call it, I had a… my cousin she is a heavy drinker! and when she lost her baby, that’s when she started drinking. Like she never drunk before but then when she just lost it was…yeah, we, we um we’re there to support her or anything but just seems like sometimes she just like pushes us away, which is really sad but we’re still, I think, [we] care, like you know, [give] love and support that she needs. So we’re just gonna be there and keep supporting her kinda thing, so…yeah” (Focus Group 9: Quote 7).

*“Oh my dad, when we had this family function, he wanted to drive the van onto the road just so that other people can park into the parking…well my brother was rolling down on…those plastic bike things and well my dad didn’t see him coming down the hill and he ran over him and that like sort of thinged off. Yeah it stuck with me yeah and that was like one of the other areas…it mucks up your judgement” (Focus Group 8: Quote 8*).

The two young people sharing these accounts reflect on how even though alcohol may give temporary relief from one’s sorrows, in their words, “it mucks up your judgement” and the situation is “really sad”. The young person’s offer of support to her cousin mentioned in Focus Group 9 (Quote 7) reflects how some of our Pacific young people might internalise responsibility not only to help develop protection strategies for the sufferer, but also for themselves whereby they may use this situation as evidence for why they should not drink or not drink irresponsibly.

A consequence of heavy drinking as attested to by a participant of Focus Group 4 is financial strife for the family. The heavy drinker in this young person’s family was his father who was responsible for paying their family bills.

*“Dad’s pretty adamant on drinking. He has to have alcohol every week and he knows that there’s mortgage to pay, there’s bills to pay and that, but he still has to have that* [drink] *and that affects us, yeah, financially as well, that money could be going to better use* [like] *my lunch. (Focus group 4: Quote 9)*

#### Family environment: “Fear makes drinking a mission”

In this sub-theme participants’ spoke about how a fear of parental or elder person reprimand does affect their drinking behaviour. This fear is such that it affected their ability to enjoy their drinking. A participant of Focus Group 21 articulates this point in Quote 10 below.

*P1* [My parents influence me] *in a very scary way coz if they find out, there’s no more existence of … it’s just a fear of the older* [people]*…*

P2: fear makes drinking a mission

*P1: So if you drink, you have to drink responsible coz then you have to come back home and be all nice and* [show] *smiles* [to] *mum and dad and make sure you smell nice…make sure you’re alcohol free. (Focus group 21: Quote 10)*

#### Family environment: Sibling relationships: “Trying to prove something”

Like the relationships of young people with their parents, their relationships with siblings can be quite influential, in both a positive and negative sense. Sibling rivalry is natural and can be quite healthy. In terms of sibling relationships acting as a protective factor against developing negative drinking habits, the message implicit in the talk of one of the participants of Focus Group 14 is that it is usually the responsibility of the older siblings to take care of their younger siblings and to realise that sometimes the younger siblings need to “prove something” and need space to do so. He says:

*“Yeah, oh,* [with me] *its affected me and my younger brother, coz he’s just turn 21, but…probably because he’s young, he’s always trying to prove something, up on his drinking and it’s sort of affected like oh, our relationship… now that [ ] we are both drinking [ ] we sort of have our little arguments and that…so I told him that I don’t want to drink with him anymore [ ] when he drinks…Like at his twenty first just recently I didn’t drink, I just helped out like with food and stuff. And then when he drinks, when I drink, I try not drink around him. Coz it’s, I think he…was probably trying to be the man, more than* [me]*, then coz I was the older brother. But yeah, that’s what’s affected in my family, just the relationship” (Focus Group 14: Quote 11).*

### Theme 2: Ethno-cultural pride and values

#### Ethno-cultural pride and values: “We’d never, like, wanna do anything that would hurt them”

The Pacific young people of our study were adamant that they did not want to drink in a way that would cause them to behave in a manner that would hurt or embarrass their families, themselves and even their churches. Moreover, being the brunt of gossip was of concern. So in talking about trying to drink responsibly or abstaining from drinking they raised the concern that their drinking behaviour did not “do anything that would hurt” those they cared for. The brief excerpt from Focus Group 12 (Quote 12) emphasises the belief, not uncommon to young people generally, that where their parents have sacrificed for them that they must avoid behaving in a way that would deliberately undermine that sacrifice.

“My parents worked really hard to look after us and we’d never like wanna do anything that would hurt them” (Focus group 12: Quote 12).

The next two excerpts are exchanges between participants within two different focus groups (Focus Groups 7 and 12). Their discussion highlights how Pacific young people internalise, monitor and rationalise their responsibility vis a vis their parents and community to protect their respective good names. There is a mix of pride and fear in the way in which the young people speak about this responsibility. The pride is implicit in the idea of not soiling a good family name and its potential for family unity. The fear is both in the fear of letting down the family, especially parents, and of the whole family being stuck with the stigma of an embarrassing or shameful event. The first of these two excerpts from Focus Group 12 (Quote 13) below emphasises the belief that one’s actions “falls back on your family”.

“P1: I think growing up in my family was sort of taught to like have responsibility, like both my parents drink but not like hard out, only on special occasions and stuff and weddings or weekends or family reunions. And like my parents always teach us like if you wanna do something you sort of have to know the consequences and stuff and that it’s like your fault if something goes wrong. And so like… I never like wanna be in a situation where I’m not in control of what I am doing or aware of what I am doing so that’s why I don’t drink coz like I don’t wanna be… I…don’t wanna be not aware of what I am doing and then like become aware that you did something stupid and then like…

P2: Yeah, you don’t wanna regret it, yeah, and so I have like…

P3: The next morning you’ll be…

P1: Yeah a big responsibility with your parents and you don’t wanna do something stupid and then like…

P3: It falls back on your family…

P1: Yeah, and like I don’t want them to go through anything like hard out, so…yeah. So there’s just times like for self responsibility and stuff, being aware that if you do drink hard out like maybe having people around you that you trust and stuff. I always have people around that I trust but I still don’t wanna be unconscious or anything” (Focus group 12: Quote 13).

The second is from Focus Group 7 (Quote 14) and re-emphasises the same point about the inextricable link between the reputation of the young person and that of his/her parents/family.. The excerpt below shows how this commonly cited Pacific adage: “everywhere you go you’re taking your parents [and community] with you”, is internalised by some of our Pacific children.

“P1: It’s like a bad impression, like you’re…like it’s not that you’re trying to impress the parents, it’s just that um you wanna save yourself from getting gossiped by others…

P2: yeah, the ladies…

P1: like the…the ladies they will be like…

P3: you don’t wanna bad image on you

P1: yeah

P4: and you don’t want your, your parents name to be shamed like, coz like my parents always tell me that everywhere you go you’re taking your parents with you

P1:…so you don’t wanna be an embarrassment to your family or church” (Focus group 7: Quote 14).

The idea of ‘carrying your family name with you’ is made even more explicitly ethnic-specific when Tongan participants of Focus Group 20 talk about how “everyone in Tonga knows everyone”, they know “your whole genealogy”, they ask “whose your father?” or “whose your mother?”, and so these young Tongan people feel an acute need to be careful how they behave because they are well aware of how it could impose shame on their families.

P1: You wouldn’t want to put your family or your family name to shame or anything. Everyone in Tonga knows everyone….

P2: Yeah, they go “whose your father?”, “oh, whose your mother?” ”oh ok”…

P1: …Are they from where? Are you from this village or this one? They know everything, you know, your whole genealogy and everything…. (Focus group 20: Quote 15)

The connection between family and ethnic community pride and values is brought together simply and explicitly by a participant of Focus Group 20 (Quote 16) who stated that: *I don’t want to be a bad drinker because that’s not paying tribute to my family and to my people (Focus group 20: Quote 16).*

#### Ethno-cultural values: *toka’i* or cultural respect

Ethno-cultural values provide ethnic groups with a value framework for personal and group conduct. They assist in providing context for why certain decisions might be made. The ethnic aspects of these values are best captured by indigenous terms. For the Tongan participants of Focus Group 20, the Tongan concept of *toka’i* was raised as important to the process of deciding whether or not to accept an invitation to drink. From the interchange between two participants of this focus group (cited at Quote 17) how these young people understand the principle of *toka’i* is illuminated somewhat. The specific mention of this concept using the indigenous term highlights that for some Pacific youth in New Zealand, the traditional values of their specific ethnic groups may still be quite strong.

P1: If someone goes ‘here’s a beer’ you drink it.

[P2: Yeah, you don’t go oh yeah and turn it down… yeah, its sign of a boundary like if you if you if someone offers you a drink and you drink it…

P1: if you decline it, then it’s an insult kind of thing.

P2: It’s not probably giving into pressure, but it’s like um, it’s called toka’i, like, um how do you say it, you’re thankful that someone will come to you and actually like it would be rude to turn them down kinda thing, if you know what I mean…

P1: It’s like, it’s like, yeah…

P2:…The way we were raised aye, you not…

P1: If someone goes ‘have a drink’ your like you know…

P2: Coz like the other night one of my cousins came from Tonga and he was like, you know I was working the next day and he was like ‘lets drink’ and I’m like ‘bro I’m working’, he goes ‘drink’, so oh yeah stuff work, we’ll go, and then I ended up going to work with a hangover, but you know, I kicked it with my cousin… It’s a cultural thing to invite someone aye…

P1: Yeah, it’s a rule, even when it comes down to food and anything like, ‘hey, ha’u ka’i’ (hey, come and eat), yeah if you’re eating and someone’s sitting here and you(‘re) like…come eat…

P1: even if you got one little sandwich, and you like hope he doesn’t come over but you still…

P2: nah, it’s rude aye. Yeah, whatever you’re got you just share it aye” (Focus Group 20: Quote 17).

#### Ethno-cultural values: “It’s not about where you drink but who you drink with”

Polynesian cultures traditionally frown on social mixing or fraternising by young males and females, especially at public forums where traditional social etiquette is prized. Increasingly, these social taboos are weakening as Pacific communities take on more western cultural values about how to conduct various male–female relationships. But for some Pacific cultures, and even in New Zealand today, as evidenced by the talk of one of the participants of Focus Group 21 below, these taboos are still recognised or upheld by Pacific families.

*“Yeah…it sucks* [about] *certain perceptions that everyone says…. In our family…you don’t see any of the girls drinking. And when there is that one girl who drinks and she was an outsider;* [she] *was my cousin’s wife, she got looked at, she got frowned upon, she got called names…. It’s not about where you drink, but who you drink with. But it’s also about the fact that boys have their own drinking spot and then she wants to come in and join in with them…it’s not a good thing, coz you never see that on our side of the family; boys and girls drinking[together] unless the first cousins are really close with us and they trust that drinking with them is safer than anywhere else…” (Focus group 21: Quote 18).*

### Theme 3: Church influences

The significance of church to Pacific young people in New Zealand is captured by the words of a participant from Focus Group 7 (Quote 19): *“Island parents, they’re high on church. They’ll get really disappointed if you don’t go to church every Sunday” (Focus group 7: Quote 19).* Here he suggests that even if a young person is not an active church goer, they are usually still aware of church and have some indirect association with it, as is the case for another participant of the study from Focus Group 14 (Quote 20) who monitors his drinking on Saturday nights because he is aware that he must take his mother to church the following morning.

*“I’m thinking like for tomorrow, usually it’s a Sunday, and mainly the most important thing is just to not drink much coz of my mum…I have to drop her off when she has to go to church the next day…so I have to slow down, I can’t drink till three in the morning. I usually stop, and then I just socialise and* [know I’ve] *had my limit” (Focus group 14: Quote 20).*

For those participants of our study who declared more active participation in church, they talked about how their church would have activities that teach them about the risks associated with alcohol drinking. Quote 19 below from Focus Group 2 makes this point.

*“There will be activities about drinking* [and] *the risks;* [we would] *gather with a video* [and] *all watch it together* [to] *see the side effects about it…; learning when you grow up, you listen, and that’s when you know* [what] *your mistakes are - in church - when you are young” (Focus group 2: Quote 21).*

For another young person from Focus Group 10 the protective value of abstinence is for him assumed because it was such a taken-for-granted part of the way they lived their lives as members of their religious community:

You just learn like not to touch alcohol, not to smoke and…you get used to it and it becomes a norm in your daily lives. (Focus group 10: Quote 22).

### Theme 4: Peer relationships and personal aspirations

#### Peer relationships: “Wake up call”

From the narratives of the Pacific young people of this study there was, particularly at high school, peer pressure to drink and experiment with alcohol. For a participant from Focus Group 13 (Quote 23) his irresponsible drinking over the years was largely attributed to his succumbing to pressure by his friends. Unfortunately, it was not until he was diagnosed with liver problems that he decided to stop. For him, to use the words of the participant cited in Quote 24 from Focus Group 18, his health problems were his “wake up call” to stop.

Um, the reason why I am not drinking or I haven’t drank in the last 2 years is because I have health problems, um liver problems. But I did drink and that was because I was peered pressured into it, um not peer pressure I just started like sipping it and tasting it and then it’s like smoking when once you smoke you can’t stop, it was like that for drinking and I started to drink and then when I found out that I had health problems I stopped so I stopped since then” (Focus Group 13: Quote 23).

*Yeah like you just get that wake up call…something happens and then you do something real stupid then…once you’re sober…your friends sort of like tell you where you were and what you did and* [that] *sort of wakes you up in a sense you* [think] *was I really that bad, did I really do that? (Focus Group 18: Quote 24)*

This “wake up call” can also take the form of realising that you are about to lose someone close to you as was the case for another young person from Focus Group 12 (cited at Quote 25).

*“…the real reason why I chose to give up is because when I was drinking I didn’t know who I was. Sometimes in the mornings I’d wake up and like wouldn’t even remember what I was doing so that kinda had an affect on myself and um like being under the influence of alcohol, you don’t um, its, it comes down to yourself you know and what you do but like um, sometimes its out of your control and you can’t do anything and its also unsafe because who knows what would happen but um I met someone in 2005 and this person told me when we first met, is that drinking is not… you know…it, its good in a way but also bad in a way because um drinking has like caused a lot of violence and problems in societies, so… I didn’t really listen; I didn’t take it into account until I knew that I was about to lose that friend. So like it was kinda like a wake up call. And like just, I think using alcohol was just, I think just to get rid of all the um burdens that I, I had. So yeah, that friend has been a lot of help to me in the past and help me overcome my drinking” (Focus Group 12: Quote 25)*.

Peer influences can be both positive and negative as the above quotes suggest. While the young people in the above quotes recognised their vulnerability to peer pressure in the negative sense, they also showed that supportive peers willing to warn them of the risks of alcohol dependency can be a positive deterrence. What is also “woken up” so to speak for these participants is the realisation that even with positive peer support they themselves are ultimately responsible for protecting themselves.

It is interesting to note that schools were spoken about mainly in terms of being the site where those who talked about peer pressure felt it. In discussing the legal drinking age one of the high school students of Focus Group 4 stated (Quote 26):

“I reckon it should be 20, coz then it takes the pressure off high school seniors and lets us focus on exams and stuff?” (Focus Group 4: Quote 26).

#### Personal aspirations: “I wanna support myself” and “Think about the time ahead”

The final sub-theme of this final theme re-emphasises the point that Pacific young people must recognise that they themselves must want to protect themselves from harm in order for any protective factor to work. This is explicit in the comments of another participant from Focus Group 4 (Quote 27) who says:

“… *in the future I wanna support myself …that’s one of the things that’s encouraging me not to drink” (Focus Group 4: Quote 27).*

Focusing on personal career aspirations can also buffer against young people developing harmful drinking habits, especially those careers that require physical prowess. This point was raised by a participant from Focus Group 2 (Quote 28) who saw the direct link between harmful drinking and decreased sporting prowess. He says:

*“When I think about like the time ahead of me…I don’t want to wreck myself coz I am a full-on sporter. I am into my sport and* [I don’t want to] *wreck it early” (Focus Group 2: Quote 28).*

Pacific young people have their work cut out for them in terms of resisting the binge drinking culture that currently characterises them and pervades their social milieu. While environmental (including social and structural) factors are important to consider in working through protective mechanisms, so too are personal factors.

## Discussion

The situation of Pacific young people in New Zealand is of interest to policymakers and service providers alike, not only for the challenge they pose as a pan-ethnic and largely diasporic population but also for the claims made by different studies that Pacific peoples have high resilience levels due to their strong social structures and support systems such as church and extended family [28-30]. This paper describes how the Pacific young people who participated in our focus groups responded to the discussion areas on perceptions and experiences of alcohol consumption, effects of drinking on family, and why they practiced abstinence or responsible drinking. While participant responses to what constituted responsible drinking for them tended to focus on displays of outward behaviour, these perceptions offer some insight into how Pacific young people link outward behaviour with what is responsible drinking and what is not.

As in other studies (although mostly quantitative surveys, for example, the Pacific Alcohol and Drug Consumption Survey [31] and Abbott et al’s Pacific Islands Families Study [32]) our study also saw some variations in responses between different specific ethnic groups within the Pacific label. Nevertheless, these variations were minimal and mostly arose when participants discussed (or not) indigenous values or practices using their indigenous language.

The results of this study showed that Pacific young people in New Zealand are strongly influenced by three main social institutions: their families, the churches their families attend and their peer groups. What is of interest here is how in the narratives of these young people the high school did not feature as highly as the other three as a specific site for thinking about protective strategies against drinking. What was discussed in relation to schools was the significant peer pressure felt by students to experiment with alcohol.

The work of Evans and Becker on parents with HIV and AIDS [33], identifies two key protective factors against harm (i.e. depression): 1. individual attributes such as problem-solving skills, high aspirations, faith and religious beliefs, positive peer relationships; and 2. family characteristics, such as having caring and supportive family relationships. In our study the experiences and thoughts of our young people suggest that both individual attributes and family characteristics as described by Evans and Becker are indeed important, but that so too were other factors such as individual and family access to wider community support networks such as church communities and extended family and peer networks. The added church, extended family and peer networks contributed to the stability and identity of their families and themselves. This is not to say that access to and engagements with these added groups were not without problems. Rather, it is to say that because the young people raised them in their talk, there is an assumption that they have some place in their lives, even if it is a constantly tense and negotiated place. As Ungar et al [34] suggest it is in the successful negotiation of those tensions that we can glean what might constitute resilience and a protective factor.

The distinction between “happy” versus “heavy” drinkers provides a barometer for gauging how Pacific young people might think about and evaluate positive or negative risk. Given the low socio-economic status of Pacific peoples in New Zealand the distinction may collapse when the drinker becomes addicted and only finds happiness when he or she is drinking.

Cultural assimilation models have suggested that eventually indigenous and migrant peoples will take on the dominant cultures and identities of their host community [35]. In New Zealand this has been replaced with models of bi and multiculturalism [35,36]. The latter of which recognises that the people’s sense of belonging to a place or culture travels with them and are kept alive through language, cultural practices, social institutions and value systems. When the Pacific young people of our study made deliberate reference, quite naturally, to (a) their practices of *toka’i,* or (b) to the social shame that would befall their parents, families and churches if they were to behave drunkenly or if they were to fraternise in mixed gender groups, or (c) to the bad feeling they would get if they were not able to take their mothers to church Sunday morning because of a heavy Saturday night of drinking, they implicitly endorsed not only the view that there still exists for them a connection with their Pacific cultural value systems, but also that they can and do hold at least bi if not multicultural identities. This multiple layered identity and sense of belonging is also evident for Pacific peoples in New Zealand whether or not they are fluent in their ethnic specific Pacific languages.

Pacific indigenous values and practices are often cited by policymakers in New Zealand as cultural touchstones significant for identifying underlying ethnic value systems and relevant to designing policy frameworks or practice guidelines [24, 37]. The narratives of the Pacific young people of this study suggest that such values are still relevant to their current contexts and co-exist with other cultural groups they may also belong to. Traditionally, Pacific cultures have privileged the voice of elders over that of youth. This has become a barrier for some youth in raising situations of abuse by parents or elders [10].

In engaging with the global community Pacific families in New Zealand and in the island nations are raising children who live in environments where they are increasingly gaining access to opportunities to speak out against abuse and to develop protective mechanisms. Understanding the ethnic and cultural nuances of what Pacific young people say are of importance to them when deciding whether or not to speak out or whether or not to drink alcohol – excessively, responsibly or not at all – is as important as understanding the clinical measures for developing prevention or harm reduction interventions for them. There is not one way, as pointed out by Ungar and his colleagues [34], in which to explain what would be a protective factor or not. It is in understanding the detail of the context in which certain events or experiences become positive and can neutralize negative risk that is the more salient analytical exercise.

## Limitations

This study is not without limitations. Firstly, the study was conducted with participants from a single urban centre; Auckland, New Zealand. This was believed to be appropriate because the majority of the New Zealand Pacific Island population live in the Auckland region [19]. Purposive sampling was used to specifically identify Pacific youth attending high school or university who self-identified as abstinent or responsible drinkers. The focus groups then sought to explore opinions about protective factors only from these two groups of youth. It is possible that similar or different factors may have been identified by Pacific youth outside of these groups. It is also possible that other protective factors exist that were not expressed by the participants of these two groups. However it was not the aim of this study to attempt to represent all opinions of Pacific youth living in New Zealand. To obtain a more generalisable data set a quantitative approach would be required in future study. Thirdly, the study adopted focus group discussions as the data collection method of choice. However, participant’s responses can be influenced by others in the group and people may be reluctant to share sensitive information in a group setting. Whilst gender specific focus groups were held, logistical constraints prevented all focus groups from gender-matching the facilitator and the focus group. Where this was the case there was a noticeable difference in the quality of sharing received from participants of a different gender to the facilitator.

## Conclusions

This study offers a qualitative analysis of the narratives offered by some Pacific young people in New Zealand on key factors that underpinned and influenced their decisions to abstain or drink responsibly. The narratives highlighted three key communities of influence on these young people: family (including siblings), peers and church. Interestingly, the school community was not discussed as much. Why this was so is an area that could be explored further in future studies. The narratives suggest that the young people of this study negotiated through these three communities of influence their decisions whether to drink alcohol, whether to drink excessively or at all and that for each young person the way in which those three communities came together to support their decisions would always depend on the specificities of their lived contexts. Understanding these specificities requires understanding the ethnic dimensions of their lives. The Pacific young people of this study, who all live in Auckland, New Zealand, share ethnic values and cultural circumstances that both connect with the traditional cultures of their parents or ancestors and with the more contemporary cultures of their churches, peers and neighbourhoods. This article concludes that Pacific young people live lives that share some things in common with other New Zealand young people and other things which are more specific to them as a Pacific ethnic group. In the development of alcohol related prevention and harm reduction strategies that seek active Pacific young person and family compliance, it is these “other ethnic things” that requires careful and more qualitative consideration.

## Competing interests

The authors declare that they have no competing interests.

## Authors’ contributions

TS and JP were involved in the study design. KS coordinated the project including ethics, data collection, analysis and writing under the supervision of TS, AW and JP. KS wrote the first draft of the manuscript and incorporated feedback from the other named authors (JP, TS, LD, AW) into the final version. TS led the revision process. All authors read and approved the final version of the manuscript.
